# RNA isolation from precision-cut lung slices (PCLS) from different species

**DOI:** 10.1186/s13104-017-2447-6

**Published:** 2017-03-09

**Authors:** Monika Niehof, Tobias Hildebrandt, Olga Danov, Kirsten Arndt, Jeannette Koschmann, Franziska Dahlmann, Tanja Hansen, Katherina Sewald

**Affiliations:** 1 Division of Preclinical Pharmacology and In Vitro Toxicology, Fraunhofer Institute for Toxicology and Experimental Medicine ITEM, German Center for Lung Research (DZL), Biomedical Research in Endstage and Obstructive Lung Disease Hannover (BREATH), Excellence Cluster REBIRTH, Nikolai-Fuchs-Str. 1, 30625 Hannover, Germany; 2 Immunology and Respiratory, Boehringer Ingelheim Pharma GmbH & Co. KG, Ingelheim am Rhein, Germany; 3 geneXplain GmbH, 38302 Wolfenbüttel, Germany; 4 Pathology Unit, German Primate Center GmbH, Leibniz-Institute for Primate Research, Kellnerweg 4, 37077 Göttingen, Germany

**Keywords:** RNA extraction, RTqPCR, Microarray, RNA quality, Lung tissue, Lung material, Ex vivo, Organotypic tissue

## Abstract

**Background:**

Functional 3D organ models such as precision-cut lung slices (PCLS) have recently captured the attention of biomedical research. To enable wider implementation in research and development, these new biologically relevant organ models are being constantly refined. A very important issue is to improve the preparation of high-quality RNA (ribonucleic acid) from PCLS for drug discovery and development of new therapies. Gene expression analysis at different levels is used as an important experimental readout. Genome-wide analysis using microarrays is mostly applied for biomarker selection in disease models or in comprehensive toxicological studies. Specific biomarker testing by reverse transcriptase quantitative polymerase chain reaction (RTqPCR) is often used in efficacy studies. Both applications require high-quality RNA as starting material for the generation of reliable data. Additionally, a small number of slices should be sufficient for satisfactory RNA isolation to allow as many experimental conditions as possible to be covered with a given tissue sample. Unfortunately, the vast amount of agarose in PCLS impedes RNA extraction according to the standard procedures.

**Results:**

We established an optimized protocol for RNA isolation from PCLS from humans, rats, mice, marmosets, and rhesus macaques based on the separation of lysis and precipitation steps and a magnetic-bead cleanup procedure. The resulting RNA is of high purity and possesses a high degree of integrity. There are no contaminations affecting RTqPCR efficiency or any enzymatic step in sample preparation for microarray analysis.

**Conclusions:**

In summary, we isolated RNA from PCLS from different species that is well suited for RTqPCR and for microarray analysis as downstream applications.

## Background

Functional 3D organ models are considered to have high impact and value in translational science. In lung research, tissue models such as precision-cut lung slices (PCLS), parenchymal strips, and isolated vessels and bronchi are used intensively and have been shown to be of importance, since their cellular composition includes varying cell types with many functions. PCLS, for example, contain epithelial cells, fibroblasts, smooth muscle cells, nerve fibers, and immune cells such as antigen-presenting cells, macrophages, and T-cells. Cells interact with each other, thereby reflecting the highly specialized function of the lung.

Precision-cut lung slices are used primarily for basic research and non-clinical efficacy and toxicity testing of (biological) compounds. The general perception has changed for many reasons. First of all, there have been significant improvements in the preparation and culture of ex vivo tissues, so that thin and precise sections are obtained, which can be maintained for days, weeks, and even months [1–3]. Today they form the basis for high quality research for drug discovery and new therapy development. In the context of efficacy and toxicity testing, they address the fact that individual cell types may respond quite differently to the same drug or substance. Therefore, lung tissue is exposed ex vivo for example to drugs, chemicals, mitogens, and bronchoconstricting agents, and is then analyzed for phenotyping of cellular changes, respiratory toxicity and efficacy, broncho- and vasoconstriction and dilation, immune responses, and tumor invasion [4–6]. Moreover, the use of human tissue is considered to be more predictive for human responses to pharmaceuticals than animal experiments. The high failure rate of drugs during clinical trials is driving the pharmaceutical industry towards translational approaches—enabling early decisions in drug development based on models, for example with human cells and tissues. To enable wider implementation in research and development, new models will be developed and existing models will be further improved. Thus, constant work is being done to refine methodologies, endpoints, and analyses. A very important issue is to improve the preparation of high-quality RNA from PCLS for drug discovery and development of new therapies. This will open up the opportunity to analyze gene expression signatures, to run microarray analyses for pattern comparison, and to get mechanistic insights into selected targets and pathways in PCLS. Preparation of high-quality RNA from PCLS from different species, however, is still a difficult issue for many researchers working with agarose-filled lung tissue. The large amount of agarose in PCLS impedes RNA extraction according to the standard procedures.

High-quality RNA, however, is an essential requirement for convincing results to be obtained in gene expression analysis using RTqPCR [7–9] or microarrays [10]. Total RNA must fulfill the following main criteria: (a) free from protein, (b) undegraded, and (c) free from enzymatic inhibitors for downstream applications [11, 12]. Therefore, RNA quality assessment includes both purity and integrity. The RNA fraction of interest for gene expression analysis is mRNA, which constitutes only a small amount (2–5%) of total RNA. The vast majority is rRNA (~80%) and it is this fraction that is generally used for quality evaluation when performing RNA extractions [13]. RNA has its absorption maximum at 260 nm and the ratio of absorbance at 260 and 280 nm is used to assess the purity of RNA preparation. Pure RNA has an A_260_/A_280_ ratio close to 2.0. An A_260_/A_280_ ratio greater than 1.8 is usually considered an acceptable indicator of good RNA quality, whereas lower levels indicate protein contamination [14]. Concerning RNA integrity highly accurate information is provided by the Bioanalyzer 2100 (Agilent) as state-of-the-art technology. It is a microfluidic capillary system using a lab-on-a-chip approach, combining capillary electrophoresis with laser-induced fluorescent detection [7, 13, 15, 16]. The system employs an algorithm that interrogates the mobility of all fractions of RNA to estimate an RNA integrity number (RIN) as quality score [13, 15, 16]. The RIN software algorithm allows the classification of total RNA based on a rating system from 1 to 10, with 1 being the most degraded and 10 being the most intact RNA [15, 16]. A RIN higher than five is recommended as good total RNA quality, a RIN higher than eight as perfect for downstream applications [7, 17]. The presence of potential contaminants that may act as enzymatic inhibitors in downstream applications can be determined by measuring the A_260_/A_230_ ratio. For pure RNA, values are around 2.0 can be expected. If the ratio is appreciably lower, it may indicate the presence of contaminants with absorbance at 230 nm, such as guanidinium isothiocyanate or phenol [13, 18, 19]. There is, however, no consensus on an acceptable lower limit of this ratio and it is presumed that the A_260_/A_230_ ratio additionally depends on RNA concentrations [18–20].

Our goal was to establish a protocol for RNA isolation from PCLS from different species that would yield RNA of high purity and integrity—even in the presence of a large amount of agarose in PCLS, which impedes RNA extraction—and is well suited for gene expression analysis as a downstream application. For validation, A_260_/A_280_ ratios, RIN numbers, A_260_/A_230_ ratios, and impact on PCR efficiency are analyzed, and quality assessment of microarrays is shown.

## Methods

### Animals

Female rats [Wistar, Crl:WI (Wu), nulliparous and non-pregnant] and Balb/c mice [nulliparous and non-pregnant] were housed under conventional and certified laboratory conditions in a regular 12-h dark/light cycle at an ambient temperature of 22 ± 2 °C and a relative air humidity of 55 ± 15%. Diet and drinking water were available ad libitum. Animals were acclimated for at least 1 week and sacrificed by an i.p. overdose (~100 mg/kg body weight) of pentobarbital sodium (Narcoren^®^, Merial GmbH, Hallbergmoos, Germany) at the age of 10–12 weeks.

Lungs of common marmosets *(Callithrix jacchus)* and rhesus macaques (*Macaca mulatta*) were obtained from German Primate Center (Göttingen, Germany). Marmosets were anesthetized using diazepam (Ratiopharm, Ulm, Germany) and alfaxalone (Alphaxan^®^, Jurox (UK) Limited, Worcestershire, United Kindom) followed by a lethal dose of pentobarbital sodium (Narcoren^®^, Merial GmbH, Hallbergmoos, Germany) under deep general anesthesia. Rhesus macaques (*Macaca mulatta*) were deeply anesthetized using a combination of ketamine (Ketavet^®^, Pfizer, New York, USA) and xylazine (Rompun^®^, Bayer, Leverkusen, Germany). The abdominal cavity was opened along the linea alba, and the abdominal aorta was cannulated for blood sampling. The aortic cannula was subsequently used to administer a lethal dose of pentobarbital sodium (Narcoren^®^, Merial GmbH, Hallbergmoos, Germany).

### Human lung tissue

Human lung lobes were obtained from male and female patients who underwent lung resection for cancer. Tumor free tissue was processed immediately on the day of resection as described below. The average age of patients was 60 ± 10 years, and 80% of them were smokers.

### PCLS preparation, cultivation, and storage

Mouse lungs were filled in situ. Rat, human, and non-human primate lungs were filled ex situ and PCLS were prepared as previously described [5, 21, 22]. Briefly, the trachea was cannulated and the lungs were filled up with 37 °C-warm, 1.5% low-gelling agarose medium solution (Sigma-Aldrich, Munich, Germany). After polymerization of agarose to gel, lung lobes were cut into 200- to 300-µm-thick slices using a Krumdieck microtome (Alabama Research and Development, Munford, AL, USA) filled with 4 °C-cold EBSS (Sigma-Aldrich, Munich, Germany). Subsequently, precision-cut lung slices were incubated in DMEM (Dulbecco’s modified Eagle’s medium/nutrient mixture F-12 Ham (DMEM, pH 7.2-7.4) with l-glutamine and 15 mM HEPES (4-(2-hydroxyethyl)-1-piperazineethanesulfonic acid) without phenol red and fetal bovine serum supplied from Gibco™ (Life Technologies/Thermo Fisher Scientific, Dreieich, Germany) supplemented with 100 units/mL penicillin and streptomycin (Lonza, Verviers, Belgium) under standard cell culture conditions (37 °C, 5% CO_2_, 100% humidity). Two PCLS were cultured together in 500 µL DMEM as described previously [21]. Different numbers of slices were pooled, as outlined in the RNA isolation section below, immediately transferred into liquid nitrogen and subsequently stored at −80 °C.

### Isolation of cells from PCLS

After preparation each PCLS was placed in 200 µL digestion solution (DMEM supplemented with 1% penicillin and streptomycin, 100 U/mL DNase I (Roche Diagnostics, Mannheim, Germany), 2.4 U/mL Dispase^®^ II (Roche Diagnostics, Mannheim, Germany), and 150 U/mL collagenase 3 (Collagenase Worthington, Lakewood, USA)) and incubated for 30 min at 37 °C on an orbital shaker (650 rpm). Afterwards, slices were passed vigorously through a cut 1-mL tip and placed in a 100-µm CellTric^®^ (Partec, Görlitz, Germany). Cells were rinsed with 600 µL ice-cold DMEM per slice, centrifuged for 3 min at 400×*g* at 4 °C, then immediately transferred into liquid nitrogen, and subsequently stored at −80 °C.

### Cell culture

The human lung epithelial cell line A549 (ATCC^®^ CCL-185™) was obtained from ATCC (LGC Standards GmbH, Wesel, Germany). Cells were routinely cultured in 75-cm^2^ flasks in Dulbecco’s modified Eagle’s medium (DMEM, Life Technologies/Thermo Fisher Scientific, Dreieich, Germany) supplemented with 10% fetal calf serum and 0.01% gentamicin at 37 °C in a humidified atmosphere containing 5% CO_2_. Cell numbers used for RNA isolation are indicated in Figs. 2a and 3b. RNA from A549 cells was used in some experiments as a quality standard for comparison.

### RNA isolation protocols

In a first step, four rat PCLS per tube were used for RNA isolation. Several commercially available kits were used for this according to the suppliers’ instructions: RNeasy Mini Kit (Qiagen, Hilden, Germany), QIAzol^®^ (Qiagen, Hilden, Germany), MagJET (Thermo Fisher Scientific, Dreieich, Germany), and MagMAX™ (Ambion™/Thermo Fisher Scientific, Dreieich, Germany). Disruption and homogenization of PCLS in the respective solutions was performed using an Ultra-Turrax^®^ (T8, IKA, Stauffen, Germany).

In a second step, optimized RNA extraction was achieved as follows: two PCLS were pooled, followed by disruption and homogenization of PCLS in 400 µL RLT lysis buffer (Qiagen, Hilden, Germany) using an Ultra-Turrax^®^. The homogenate was transferred to 1 volume of phenol/chloroform, carefully shaken for 30 s, and centrifuged for 5 min at 12,000×*g*. Subsequently, 1 volume of chloroform/isoamyl alcohol was added, again carefully shaken for 30 s, and centrifuged for 5 min at 12,000×*g*. The aqueous phase was transferred and RNA was cleaned up with MagMAX™ magnetic beads including the spin procedure step according to the supplier’s instructions. Total RNA was dissolved in RNase-free water and stored at −80 °C. For some samples an additional clean up step was performed using the RNeasy Mini Kit clean up protocol (Qiagen, Hilden, Germany).

RNA from A459 cells was isolated with the commercially available RNeasy Mini Kit according to the supplier’s instructions.

### RNA measurements, quality control and quality criteria

RNA concentration (A_260_) and purity (A_260_/A_280_ ratio) were measured by spectrophotometry (NanoDrop 1000 Spectrophotometer, version 3.7, Thermo Fisher Scientific, Dreieich, Germany). RNA integrity (RIN) was evaluated using an Agilent 2100 Bioanalyzer^®^ (Agilent Technologies, Ratingen, Germany).

### Quantitative real time RT-PCR analysis (RTqPCR)

Reverse transcription (RT) of RNA was performed using TATAA GrandScript cDNA Supermix (A103a/A103b, TATAA Biocenter, Gothenburg, Sweden). SYBR Green (TATAA SYBRGrandMaster Mix ROX (TA01-1875R, TATAA Biocenter, Gothenburg, Sweden) was used as a fluorescent dye to determine the amplified PCR products after each cycle. The following primer pairs were used: B2M (NM_004048.2), fwd: GAGGCTATCCAGCGTACTCCA, rev: CGGCAGGCATACTCATCTTTT, 248 bp, (Invitrogen/Thermo Fisher Scientific, Dreieich, Germany); MUC5AC, #qHsaCID0017663, 144 bp (BioRad, Munich, Germany). qPCR conditions were as follows: 30 s 95 °C; 5 s 95 °C/30 s 60 °C, for 40 cycles; and 15 s 95 °C/1 min 60 °C/30 s 95 °C for the melting curve. At the end of each extension phase, fluorescence was recorded and at the end of a run quantification cycles (Cq) were determined for each sample. Serial dilutions of RT reactions (A549 for B2M, human PCLS for MUC5AC) were prepared in triplicate and samples were analyzed by qPCR to measure the Cq values. A plot of Cq values versus the logarithm of target concentrations resulted in standard curves, which were used for efficiency calculations (10^−(1/slope)^ − 1, corresponding to 100%) [23–25]. To calculate the efficiency for several individual RNA samples, RNA was transcribed to cDNA (complementary DNA), two dilution steps within the log-linear portion of the standard curve separated by factor 10 were prepared (1:5, 1:50), and Cq values were measured. The Cq difference represents the slope, which was then used for efficiency calculation.

### Transcriptome analysis

Microarray analysis was performed with Affymetrix GeneChip^®^ Human Genome U133 Plus 2.0 Arrays, using 250 ng RNA as input. All steps were performed according to the manufacturer’s instructions for the GeneChip^®^ platform (3′IVT PLUS Reagent Kit, Affymetrix, Santa Clara, USA). The steps included first-strand cDNA synthesis, second-strand cDNA synthesis, synthesis of labeled cRNA by in vitro transcription, purification of labeled cRNA, fragmentation, array hybridization, washing, staining, and final scanning of the arrays using the GeneChip^®^ Scanner 3000 7G.

To examine the quality of the microarrays before and after normalization, we used the bioconductor package arrayQualityMetrics 3.24.0 [26] under R version 3.2.1 together with R Studio [27]. For quality assessment two different automatically created HTML reports were interpreted. The results included between-array comparisons, array intensity distributions, variance mean dependence, and finally the individual array quality. Outlier detection considering the distances between array comparisons was performed by finding arrays for which the sum of the distances from all other arrays was exceptionally large. Outlier detection for the array intensity distributions was performed by computing the Kolmogorov–Smirnov statistic Ka between distributions of intensity values of all samples in each array and the distribution of the values in the pooled data. The threshold was determined to be 0.0249. Regarding the individual array intensity, the mass of the distribution in an MA plot is expected to be concentrated along the M = 0 axis with no trend in M (log ratios) as a function of A (mean average). Outliers were detected by computing Hoeffding’s statistic Da on the joint distribution of A and M for each array. The normalized data were imported into the geneXplain platform (www.genexplain-platform.com) to perform a principal component analysis (PCA). The geneXplain platform was further used to detect differentially expressed genes for all samples with the EBarrays workflow.

## Results

### Establishment of an optimized protocol for RNA isolation from PCLS from different species

For the starting experiments, we pooled four rat PCLS and used a column-based standard RNA isolation protocol (RNeasy Mini Kit). Unfortunately, this standard isolation procedure did not yield intact RNA (Table 1). We assumed that saturation of PCLS with agarose might interfere with RNA extraction using a column-based isolation procedure. To determine the amount of RNA to be expected, we isolated intact cells from fresh slices and subsequently isolated RNA using the column-based standard protocol. We obtained about 1 µg RNA of good quality with an A_260_/A_280_ ratio of around 2.0 and a RIN value above 7.0 (Table 1). Isolation of intact cells is feasible only from fresh slices; however, comfortable handling of RNA isolation in different experimental approaches requires initial storage of PCLS at −80 °C. Therefore, we tried several other commercial RNA isolation kits based on diverse procedures. Using the monophasic lysis reagent QIAzol, followed by precipitation and column purification, the resulting RNA did also not meet the quality criteria (Table 1). A different kind of RNA isolation is achived by combining a monophasic lysis reagent with magnetic-bead purification. Using MagJET or MagMAX™ beads and following the protocol according to the supplier’s instructions, only the MagMAX™ beads yielded good RNA quality, with an A_260_/A_280_ ratio of around 1.95 and a RIN value above 8.0 (Table 1).

**Table 1:** Comparison of commercially available RNA isolation kits for use with PCLS

Material	Kit	RNA yield (ng)		Absorbance 260/280		RIN	
		Avg.	Min.	Avg.	Min.	Avg.	Min.
Freshly isolated cells from rat PCLS	RNeasy Mini Kit, Qiagen	1146.5	855.0	2.04	1.99	7.8	7.4
Rat PCLS stored at −80 °C	RNeasy Mini Kit, Qiagen	<100	N/A	N/A	N/A	N/A	N/A
Rat PCLS stored at −80 °C	QIAzol^®^, Qiagen	413.3	127.8	1.64	1.37	2.7^a^	N/A
Rat PCLS stored at −80 °C	MagJET, ThermoFisher	737.0	312.3	2.21	1.94	5.8	N/A
Rat PCLS stored at −80 °C	MagMAX™, Ambion/ThermoFisher	1527.4	601.5	1.95	1.78	8.9	8.4

Results represent the means of at least three samples

*N/A* not available

^a^N/A data were calculated as RIN zero (4 out of 6 samples)

**Fig. 1:**
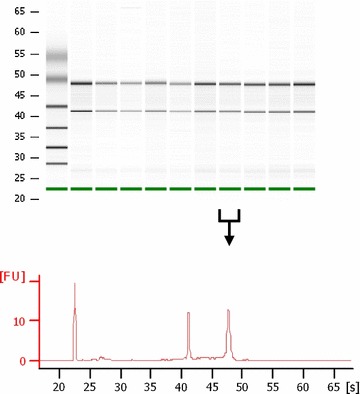
Assessment of RNA quality for human PCLS. Representative bioanalyzer results showing the quality of RNA isolated from 10 different human PCLS samples (virtual RNA gel format and electropherogram depicting fluorescence units versus run time in seconds)

To optimize RNA yield and quality we modified the MagMAX procedure by dividing the first step (monophasic lysis reagent) into a separate lysis procedure followed by conventional phenol–chloroform–isoamyl alcohol extraction (see “Methods”/“RNA isolation protocols” sections). This procedure allows a yield of at least 1 µg RNA to be isolated from two pooled PCLS from humans, rhesus monkeys, marmosets, rats, and mice (Table 2). RNA was of good quality, as evaluated by the A_260_/A_280_ ratio (around 1.9) and by RIN values (around 8.0). Additionally, an incubation period of 3 days, which is commonly used in several experimental approaches with PCLS, had no negative impact on RNA quality (Table 2). An example of RNA quality assessment for human PCLS is shown in Fig. 1.

**Table 2:** RNA isolation from PCLS from different species using an optimized protocol

Species	Incu-bation period	RNA yield (µg)		Absorbance 260/280			RIN value		
		Mean	Min.	Mean	Min.	p value	Mean	Min.	p value
Human (n = 12)	w/o	1.12 ± 0.44	0.62	1.91 ± 0.07	1.79		8.9 ± 0.7	7.8	
Human, donor 1 (n = 12)	3 days	2.34 ± 1.03	1.34	1.90 ± 0.04	1.81	0.9107	8.7 ± 0.3	8.1	0.2691
Human, donor 2 (n = 12)	3 days	1.52 ± 0.15	1.29	1.91 ± 0.09	1.75	0.4783	9.0 ± 0.4	8.6	0.7983
Human, donor 3 (n = 12)	3 days	0.98 ± 0.28	0.56	1.88 ± 0.11	1.77	0.9701	9.0 ± 0.5	8.4	0.8112
Rhesus (n = 5)	w/o	2.02 ± 0.37	1.58	1.91 ± 0.06	1.87		8.2 ± 0.5	7.4	
Marmoset (n = 5)	w/o	1.37 ± 0.16	1.16	1.87 ± 0.07	1.79		8.2 ± 0.3	7.8	
Rat (n = 5)	w/o	3.80 ± 0.78	2.48	1.95 ± 0.04	1.91		8.3 ± 1.0	6.8	
Mouse (n = 5)	w/o	5.67 ± 0.61	4.65	1.95 ± 0.01	1.93		8.2 ± 0.5	7.3	

The average is indicated ± STABW. Student’s t-test was performed to compare absorbance 260/280 and RIN values with and without an incubation period, p-values are indicated

*n* indicates the number of analysed samples, *w/o* without

### Relationship between RNA yield and 260/230 absorbance

**Fig. 2:**
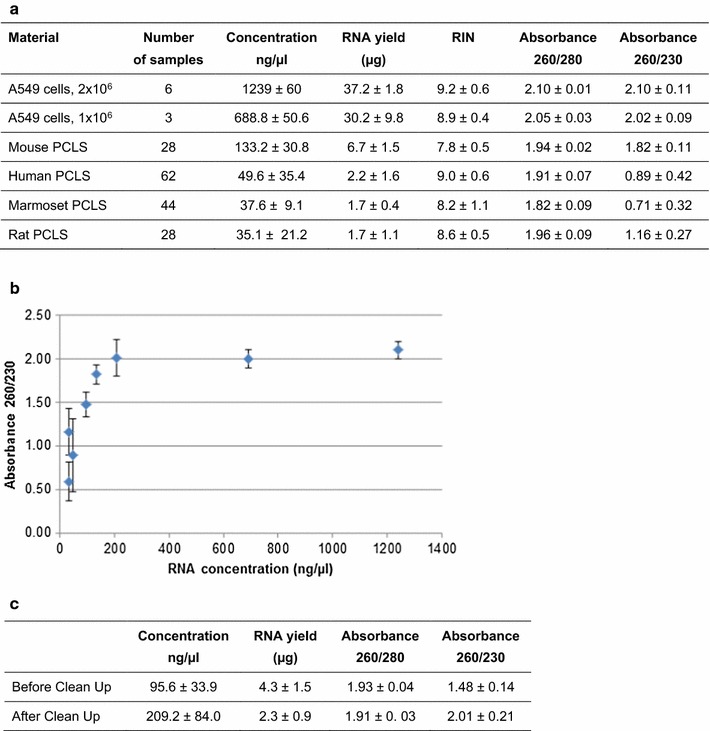
Relationship between RNA yield and 260/230 absorbance. **a** Concentration, yield, RIN, and absorbance ratios of RNA from A459 cells and from PCLS from different species (two slices each). **b** Correlation between RNA concentration and absorbance ratio 260/230. **c** RNA yield and absorbance ratio prior to and after the clean up procedure. Results represent the means of ten human PCLS samples

Quality parameters were evaluated in over 20 different isolation experiments per species (human, marmoset, rat, mouse). For all samples tested, the A_260_/A_280_ ratio and the RIN values met the requested criteria, with A_260_/A_280_ ratios >1.8 and RIN values >7 (Fig. 2a). A_260_/A_230_ ratio differed between samples. For human, marmoset, and rat PCLS an A_260_/A_230_ ratio much lower than 2.0 was measured, whereas for mouse PCLS as well as for different preparations of A549 cells used as quality standard the A_260_/A_230_ ratio was around 2.0 (Fig. 2a). With the same slice size mouse PCLS contain greater cell numbers per slice than other species. Therefore, RNA isolation from mouse PCLS resulted in a higher RNA concentration than with other species. The data indicate that the A_260_/A_230_ ratio in the preparations strongly depends on the RNA concentration of the sample, as low RNA concentrations of <100 ng/µL resulted in A_260_/A_230_ ratios <2.0 (Fig. 2b). Introducing an additional clean-up step resulted in higher concentrations and improvement of the A_260_/A_230_ ratio to around 2.0 (Fig. 2c). However, this step further substantially reduced the already small RNA yield (Fig. 2c), and, therefore, restricted the number of possible downstream applications. As we prepared all samples according to the same protocol, we assumed that they would all contain the same amount of contaminants absorbing at A_230_ despite the different A_260_/A_230_ ratios. In this case, the low ratio would solely depend on the concentration of RNA, while the amount of contaminants that would be transferred to downstream applications might not be different. PCR is extremely sensitive to impurities such as salts, phenol, chloroform, and EDTA, which is why we subsequently tested wether different A_260_/A_230_ ratios of our samples would affect RTqPCR analysis.

### RTqPCR as a follow-up endpoint for RNA from human PCLS

**Fig. 3:**
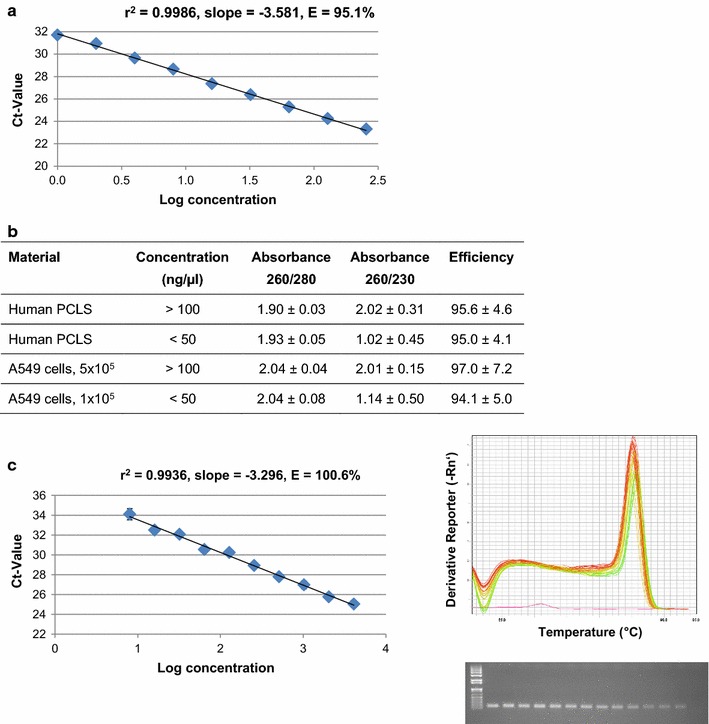
RTqPCR as a follow-up endpoint of RNA from human PCLS. **a** Efficiency calculation for B2M primer using several A549 cDNA dilutions. **b** Efficiency analysis for different RNA preparations from A549 cells and human PCLS (different donors). Results represent the means of at least three samples (RIN > 7.0). **c** MUC5AC expression in human PCLS as an example of lung-specific gene expression (calibration curve, melting curve, gel image)

Potential inhibition of PCR by contaminants can be evaluated by analyzing the effect on amplification efficiency. Therefore, we established a standard curve with A549 RNA as a quality standard (A_260_/A_280_ = 2.09, A_260_/A_230_ = 2.12, RIN = 9.3) for the reference gene B2M. Several cDNA dilutions were used, starting with a cDNA equivalent to 50 ng RNA. The resulting calibration curve indicates an amplification efficiency of 95.1% for this specific A549 RNA preparation (Fig. 3a). For efficiency comparison, we tested RNA samples with low concentrations (below 50 ng/µL) and a low A_260_/A_230_ ratio and RNA samples with higher concentrations above 100 ng/µL and an A_260_/A_230_ ratio around 2.0, from human PCLS and A549 cells, respectively (Fig. 3b). The results show that PCR amplification efficiency was around 95% for all samples, independent of the A_260_/A_230_ ratio (Fig. 3b). These data support the hypothesis, that the total amount of contaminants with absorbance at 230 nm that is transferred to the RTqPCR seems to be low and might be disregarded in our preparations. The low A_260_/A_230_ ratio is only a result of the low RNA concentration, so that further clean-up steps, improving the ratio but at the same time strongly reducing the total amount of RNA, are unnecessary. Finally, we analyzed the expression of an airway-specific gene. MUC5AC is one of the polymeric mucins forming the airway mucus gel under normal physiological conditions, with overproduction in chronic airway diseases [28]. The standard curve is linear (r^2^ = 0.9936) and indicates an amplification efficiency of 100.6%, the melting curve shows a single peak, and the gel image shows a single band for each dilution (Fig. 3c). In summary, applying our optimized RNA extraction protocol, RNA preparations from PCLS are well suited for RTqPCR as a downstream analysis.

### Genome-wide gene expression analysis as a follow-up endpoint for RNA from of human PCLS

Finally, we verified wether RNA isolated from PCLS samples can be used for microarray analysis. For this evaluation, we used RNA from PCLS from three human donors 3 days after treatment with different chemicals (1 control and 11 different treatments, n = 36 samples in total, A_260_/A_280_ absorbance ratio = 1.90 ± 0.08, RIN = 8.9 ± 0.4).

Firstly, we evaluated wether low A_260_/A_230_ ratios of RNA from human PCLS affect the yield of cRNA using the 3′IVT PLUS Reagent Kit (Affymetrix, Santa Clara, USA). Here again, we used A549 RNA with an A_260_/A_230_ ratio of 1.9 as a quality standard for comparison (Table 3). Due to low RNA concentrations (see above), the human PCLS RNA preparations have much lower A_260_/A_230_ ratios (Table 3). In microarray experiments, 250 ng RNA resulted in the same yield of cRNA in all samples (Table 3). These results indicate that the RNA from PCLS contained no contaminants that might have interfered with any enzymatic step during the Affymetrix protocol.

**Table 3:** cRNA yield after sample preparation with 3′ IVT PLUS Reagent Kit (Affymetrix)

Material	Absorbance 260/230	Yield cRNA (µg)
A549 cells	1.90 ± 0.17	58.6 ± 15.6
Human PCLS	0.60 ± 0.21	51.5 ± 12.1

250 ng RNA were used as starting material. Results represent the means of 20 samples, all samples with A_260_/A_280_ ratios around 1.9 and RIN values above 8.0

**Fig. 4:**
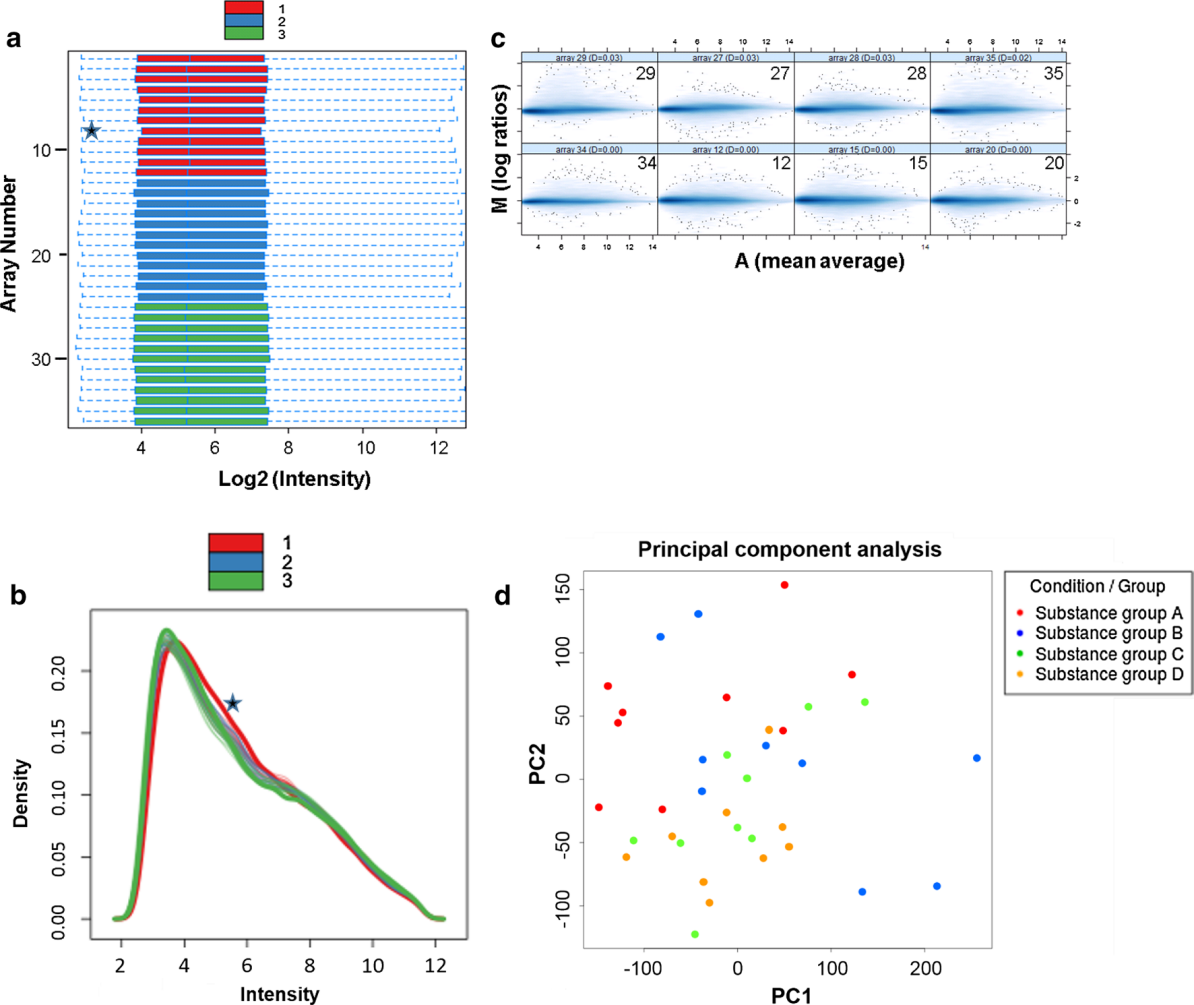
Genome-wide gene expression analysis as a follow-up endpoint of RNA from human PCLS. Bioinformatic quality assessment of 36 microarrays. Legend color *red* belongs to donor 1, *blue* to donor 2, and *green* to donor 3. **a** *Box plots* of array signal intensity distributions, where each box corresponds to one array. Signal intensities are in log2 scale (after RMA normalization). Medians are shown as a *blue line* within the *boxes*. One array (array 8) exceeded the threshold; it was considered an outlier and marked with an asterisk. **b** Density histograms of the microarrays. Signal intensities are in log2 scale (after RMA normalization). The curves of the individual donors are superimposed. Density distribution of one array (array 8, donor 1), marked with an *asterisk*, shows a shift to the right. **c** Eight MA plots of normalized gene expression data. The figure shows the four highest values of Hoeffding’s statistic and the four lowest ones. Da values are given in the Ma plot headers. No array showed a Da > 0.15, and none were marked as outliers. **d** Principal component analysis of four different substance groups (**a**–**d**). The two-dimensional *scatter plot* shows homogeneity of all groups for the first two principal components

Secondly, we performed quality assessment of the microarrays. Using the bioconductor package arrayQualityMetrics, we did not detect a high number of outlier arrays that might have been due to problems in the experimental procedure. Only two outliers (arrays 8 and 31) were identified after background correction and normalization in the section about distances between array comparisons, drawn with a false color heatmap (data not shown). Figure 4a shows the array signal intensity distributions of the RMA normalized data and represents summaries in box plots. The boxes of the signal intensity distribution box plot have similar positions and widths. Only array 8 shows a slide shift, indicating background signals. Figure 4b presents density histograms of the microarrays. The curves of the individual donors are superimposed. There was no reduction of the right tail, which would have indicated a lack of signals, nor a prominent bulge at the upper end of the intensity range, indicating signal saturation. Only array 8 shows a slide shift, indicating background signals. Figure 4c shows eight MA plots to figure out the individual array quality. Concerning the individual array intensities we could not observe arrays with different background intensities (trend in the lower range of A) or a saturation of signals (trend in the upper range of A). PCA is used to project the multivariate data vector of all substance groups into a two-dimensional plot. Figure 4d shows a scatterplot of items of four different substance groups at their transformed coordinates according to the first two principal components. No clustering of substance groups was observed during PCA, which is a good basis for further downstream analyses [29]. Due to the good RNA quality, we were able to detect differentially expressed genes for all samples.

## Conclusion

The large amount of agarose in PCLS impedes RNA extraction, making it difficult to obtain RNA of high quality from lung tissue ex vivo. We used several standard procedures and finally established a protocol for RNA isolation from PCLS from humans, rats, mice, marmosets, and rhesus macaques. The method is based on the separation of lysis and precipitation steps, and an additional magnetic-bead clean-up procedure. The resulting RNA is of high purity, possesses a high degree of integrity, and is well suited for RTqPCR and microarray analyses as downstream applications.

## Abbreviations

Cq: quantification cycle
DMEM: Dulbecco’s Modified Eagle Medium
EBSS: Earle’s Balanced Salt Solution
PCA: principal component analysis
PCLS: precision-cut lung slices
RIN: RNA integrity number
rpm: rounds per minute
qPCR: quantitative polymerase chain reaction
RT: reverse transcriptase
